# Phase angle and COVID-19: A systematic review with meta-analysis

**DOI:** 10.1007/s11154-023-09793-6

**Published:** 2023-03-24

**Authors:** Isabel Cornejo-Pareja, Isabel M. Vegas-Aguilar, Rocío Fernández-Jiménez, Cristina García-García, Diego Bellido-Guerrero, Francisco Tinahones, Jose Manuel García-Almeida

**Affiliations:** 1 Department of Endocrinology and Nutrition, Virgen de la Victoria Hospital, Malaga University, 29010 Malaga, Spain; 2 Instituto de Investigación Biomédica de Málaga (IBIMA), Virgen de la Victoria University Hospital, 29010 Málaga, Spain; 3 Centro de Investigacion Biomedica en Red de la Fisiopatología de la Obesidad y Nutricion (CIBEROBN), Instituto de Salud Carlos III (ISCIII), 29010 Malaga, Spain; 4 Málaga University, 29010 Málaga, Spain; 5 Department of Endocrinology and Nutrition, Complejo Hospitalario Universitario de Ferrol, Ferrol, La Coruña Spain; 6 Department of Endocrinology and Nutrition, Quironsalud Málaga Hospital, 29004 Málaga, Spain

**Keywords:** COVID-19, SARS-CoV2, Mortality, Phase angle, Vectorial bioimpedance, Bioelectrical impedance, Body composition, Length of stay, Sarcopenia, Malnutrition, Dysphagia

## Abstract

Phase angle (PhA) has been identified as a poor prognostic factor in patients with COVID-19. This study aimed to achieve a systematic review, where we discussed the potential role of PhA value as a prognostic marker of adverse clinical outcomes such as mortality and complication in hospitalized with SARS-CoV2 infection and established the strength of recommendations for use. A systematic literature review with meta-analysis was done in the main electronic databases from 2020 to January 2023. The selected articles had to investigate adverse consequences of the COVID-19 population and raw bioimpedance parameters such as PhA and published in peer-reviewed journals. GRADE tools regarded the quality of the methodology. The review protocol was registered in PROSPERO. Only eight studies, 483 studies, were eligible for the analysis. In general, differences in PhA were seen between the comparative study groups. Patients with a low PhA experienced poor outcomes. A low PhA was associated with a significantly increased mortality risk [RR: 2.44; 95% CI (1.20–4.99), p = 0.01; I2 = 79% (p = 0.0008)] and higher complications risk [OR: 3.47, 95% CI (1.16 – 10.37), p = 0.03; I2 = 82% (p = 0.004)] in COVID-19 patients. Our analysis showed four evidence-based recommendations on the prognostic value of PhA with two strong recommendations, one of moderate and another of low-moderate quality, for predicting mortality and complications, respectively. We recommend using PhA as a prognostic marker for mortality and complications in this population. Although the results are promising, future studies must identify the PhA cut-off to guide therapeutic decisions more precisely.

**Registration code in PROSPERO:** CRD42023391044

## Introduction

The outbreak of Coronavirus Disease 2019 (COVID-19) due to severe acute respiratory syndrome coronavirus 2 (SARS-CoV-2) infection has caused a global pandemic with a substantial spread of the infection and death [1]. This disease is associated with more than 660 million cases confirmed worldwide and more than 6.7 million deaths from march 2020 until January 2023 [2]. COVID-19 is a respiratory disease with high clinical variability, having identified specific clinical risk factors related to life-threatening illness, including comorbidities, older age, male gender, and host genetic variants (e.g., type I interferon auto-antibodies) [3–6].

COVID-19 patients may develop acute respiratory, nervous and musculoskeletal symptoms, leading to complications like sepsis, acute respiratory distress syndrome, thromboembolic events, coagulopathies, renal or cardiac failure, and even systemic organ failure. Therefore, it is essential to adapt support therapies such as intensive mechanical ventilation (IMV), precise fluids management, corticosteroids, and anticoagulant vasopressor treatments to care COVID-19 [7–9].

In the subacute phase, some patients may develop post-viral sequels or complications such as gastrointestinal symptoms, dysphagia, decreased food and protein intake, malabsorption, inflammation, low vitamin D levels, anabolic resistance, malnutrition, sarcopenia, fatigue syndrome or “long COVID” [10–12].

Researchers continue to identify prognostic factors for morbidity and mortality of SARS-CoV-2, focusing on both blood biochemical, drug treatments (angiotensin-converting enzyme inhibitors and/or angiotensin receptor blockers), the basic clinical variables (oxygen (O_2_) saturation, temperature, heart rate) [3, 5, 6, 13], as well as, tools for assessing body composition (malnutrition, sarcopenia, obesity or excess fat mass overhydration states) and the individual's cellular health status [14–16]. Thus, a more comprehensive insight into COVID-19 has enabled early diagnosis, stratification of disease severity, identification of potential sequelae, and an individualized therapeutic approach to patient management based on the severity of the disease [17].

In patients with severe COVID-19, malnutrition is often uncovered because of a direct effect of the virus resulting in systemic organ failure, long hospitalization, or intensive care unit (ICU) stay with prolonged immobilization [18]. Bioelectrical impedance (BI) assesses a patient´s nutritional status and body composition. Mainly, the BI measured using 50 kHz phase-sensitive devices uses whole-body measurements to classify and monitor hydration and cell mass without the use of multiple regression equations, instead focusing on raw bioelectric parameters, such as resistance (R) and reactance (Xc) [19, 20]. Resistance (R) is the opposition of the flow of low-level alternating current due to ionic fluids, and reactance (Xc) is the delay of current entry into cells related to cell membranes and cell interfaces. Phase angle (PhA) describes the lag between voltage and current and characterizes fluid distribution between the extracellular and intracellular compartments (E/I) [19, 20].

Thus, PhA is a cellular health biomarker that discloses the malnutrition and inflammatory status which can accompany these acute and/or serious disorders. PhA is a unique predictor of mortality in diverse clinical conditions [19, 21], including SARS-CoV2 infection and a potentially helpful screening tool for prognosis [22, 23]. Some studies have reported its association with poor outcomes, such as length of stay (LOS), mortality, or the need for intensive support therapies [21, 24–26]. However, the routine use of PhA to assess hospitalized COVID-19 patients has not been established due to the lack of a focused evaluation of clinical findings.

This systematic review of the literature with meta-analysis aims to establish the clinical value of PhA as a prognostic marker of adverse clinical outcomes such as mortality and complication/sequelae in hospitalized with SARS-CoV2 and to establish the strength of recommendations intended for use by health systems in clinical practice guidelines.

## Methods

### Methodology and protocol registration

This study was prepared using the recommendations of the preferred reporting items for systematic reviews (PRISMA) guide [27]. The review protocol was registered on PROSPERO with the number: CRD42023391044. Additionally, the quality of the evidence of the present systematic literature review was evaluated with the GRADE methodology (Grading of Recommendations Assessment, Development and Evaluation) [28] to develop evidence-based recommendations. The authors developed the clinical questions that guided the literature search and the requests using the PICO (Patient, Intervention, Comparison, Outcome) framework [29].

### Literature search

We conducted our systematic literature identifying potential studies with a comprehensive search in MEDLINE or PubMed, Scopus, Embase, and Web of Science databases (from database inception to January 2023) to identify studies addressing the PhA evaluation and COVID-19. A combination of the following medical subject headings and keywords was used in the title, abstract or keywords fields: “SARS-CoV2,” “COVID,” “COVID-19” AND “bioelectrical impedance”, “BIA”, “bio-impedance”, “phase angle”, “PhA”, to identify the main bioimpedance parameters together with the population of interest. The additional search terms for primary outcomes are mortality, length of hospitalization, and complications, such as sarcopenia, malnutrition, dysphagia and invasive mechanical ventilation. Also, the hand-searching of databases was completed by two authors. Articles published in English or Spanish were selected for critical synthesis, and only significant associations are reported.

### Literature inclusion and exclusion criteria

The clinical questions that guided the literature search and the recommendations were developed by the scientific committee composed of four authors with experience in body composition study and COVID-19 (I.C.-P.; J.M.G.-A.; D.B.-G. and F.J.T.) To determine the eligibility criteria, the PICO strategy [29] was adopted: in which "P" (patients), corresponding to COVID-19 patients of all genders and ethnicities; “I” (intervention), was designated as bioimpedance assessment with phase angle, “C” (comparison), was defined as altered results vs normal phase angle results, “O” (outcomes), was the mortality, LOS, severity disease or short- and long-term complications or sequels.

Exclusion criteria were as follows: (i) articles did not include a full-text description of the study; (ii) not in Spanish or English language; (iii) differences in phase angle are not evaluated regarding outcomes (e.g., mortality, length of stay, severe disease, complications or sequels); (iv) studies not published in peer‐reviewed journals; (v) meta‐analyses, reviews, protocols, case series or reports, editorials, and letter to the editor; (vi): pregnant or lactating women studies; and (vii) studies using animal models.

### Study selection

The selection process was conducted by four independent authors (I.C.-P., J.M.G.-A., D.B.-G. and F.J.T.). The reference lists of all included studies were hand-searched for missing publications. Three authors (I.C.-P., J.M.G.-A. and D.B.-G.) independently screened the selected articles for eligibility after testing the abstract and full text. Differences of opinion while selecting the articles were resolved by consensus between authors. One author (F.J.T.) reviewed each opinion difference and decided for inclusion or exclusion in this study.

### Risk of bias assessment

The methodological quality assessment of the studies was achieved using the GRADE methodology [28]. The GRADE method to provide is a standardized tool for rating the quality of evidence and grading the strength of recommendations. Many organizations have endorsed GRADE method to decrease the risk of bias, inconsistency of results across studies, indirectness of evidence, imprecision and publication bias [30]. It proposes specific criteria that should be considered, particularly in observational studies [31].

GRADE’s four categories of quality of evidence (high, moderate, low, very low) imply a gradient of confidence in estimates of the effect of a diagnostic test strategy on patient-important outcomes. This GRADE approach examines methodological quality by analysing the studies potential limitations, focusing on aspects such as study design, risk of bias, directness, indirectness outcomes, patient populations, diagnostic tests, comparison tests, indirect comparisons, inconsistency in study results and imprecise evidence. The GRADE approach to grading the quality of evidence and strength of recommendations for diagnostic tests provides a comprehensive and transparent approach [28].

The authors reviewed the literature, selecting outcomes from the studies, rating their importance, and evaluating outcomes across studies. Then the evidence profile tables for outcomes were created, including a rating of the quality of the evidence, using GRADEpro GDT software (https://gradepro.org). The tables include outcomes, number of studies, study design, risk of bias, effect, quality of evidence, and importance. The overall quality of evidence was rated across outcomes based on the lowest quality of critical outcomes. The authors then made recommendations for each topic based on the literature findings and balancing consequences (e.g., benefits/harms, values, preferences, feasibility).

### Data analysis, processing and data synthesis

The authors manually included the selected articles in a Microsoft Excel table. This Excel document contains the characteristics of selected articles, such as first author, study country, study design, comparative groups, number and type of participants [general or ICU hospitalized patients] participants characterization [sex and age], measurement time, follow-up time, BI device [and frequency (kHz)], PhA value and reference values, raw bioelectrical parameters (R and Xc) nutrition status [fat-free mass (FFM), skeletal muscle mass index (SMI), soft lean mass (SLM), skeletal muscle mass (SMM), appendicular muscle mass index (AMMI), hydration status [extracellular water/total body water (ECW/TBW) ratio, TBW/FFM] and reference value, outcomes [mortality, LOS, complications or sequels], results [(number of events/total population) ratio and effect rate [95% confidence interval (CI)], and conclusions.

Tables summarize data, which were grouped by similar categories to allow comparisons among studies. Likewise, an analysis was carried out following the GRADE methodology to evaluate the quality of the studies and develop recommendations for clinical application of PhA.

### Statistical analysis

The meta-analysis used Review Manager 5.3 statistical software. Risk Ratio (RR) or Odds Ratio (OR) and 95% CI were used for continuous binary variables, respectively. If the heterogeneity test revealed p ≤ 0.05 or I^2^ > 50%, we concluded that the index is statistically different between the studies, and the Random Effects Model (Random) is used. If the heterogeneity test p > 0.05 and I^2^ < 50%, it indicates that there is no statistical difference in this indicator between studies, and the Fixed Effects Model (Fixed) is used for merging.

## Clinical characteristics of bioelectrical phase angle

**Fig. 1:**
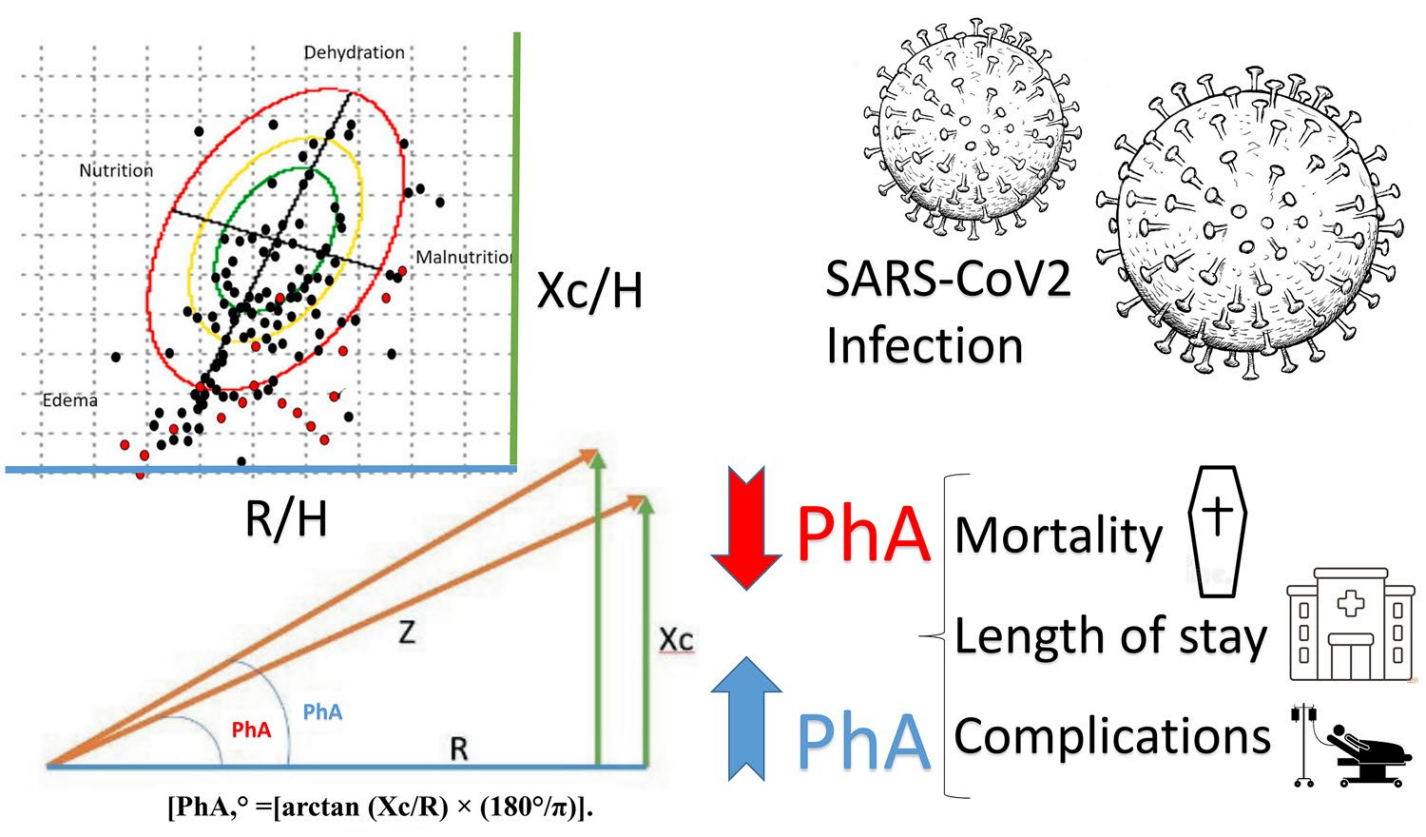
Clinical outcomes related to Phase Angle on COVID-19 patients (R: resistance; H: height; Xc: reactance; PhA: phase angle)

Bioimpedance analysis (BIA) is an indirect method to measure body composition based on the human body’s ability to conduct electricity. The current is transmitted through liquids and electrolytes, while fat and bone are not conductors [32]. Through raw impedance parameters, such as R and Xc, the PhA can be obtained: PhA (°) = [arctan (Xc/R) × (180°/π)]. By definition, PhA is positively associated with tissue Xc, as related to cell mass, integrity, and function, and negatively associated with R, which depends mainly on the degree of tissue hydration [33] (Fig. 1).

PhA is measured directly with BIA, which is fast, portable, non-invasive, reproducible, and sensitive. In addition, it has been considered a valuable tool in various clinical situations. In healthy subjects, the PhA oscillates between 5° and 7°. A low PhA (< 4°) indicates unbalanced proportion between cells and fluids. Normality curves have been established for different populations. Age, body mass index (BMI), sex, and race influence PhA values among healthy individuals. In this way, although the absolute value of the measure is a parameter that must be assessed, the standardized PhA is obtained by adjusting the PhA obtained by age and sex variables and represents an additional crude measure. That is, this value is important because it allows comparison with respect to healthy population references with the same characteristics (age and sex).

PhA is considered a reliable indicator of cell integrity and has been proposed as a nutritional status marker for adults and children after findings in numerous pathologies. It has also been proposed as a useful prognostic marker for several clinical conditions. In clinical practice, the determination of PhA allows the characterization of a patient relative to a healthy group and facilitates the follow up in the clinical care of patients at nutritional risk, such as screening and to evaluate prognosis and mortality in very diverse pathologies (HIV, cancer, anorexia, liver cirrhosis, haemodialysis, short bowel, cardiac pathology, lung disease, surgery, neurological pathology, surgical pathologies, geriatric diseases, hospitalized patients, critical patients, infectious pathologies such as SARS-CoV2, etc.) [21]. A recent meta-analysis reported normality curves and population percentiles derived in a sample of more than 250,000 patients; in general, lower levels of PhA suggest a worse prognosis and greater morbidity and mortality [21].

Other aspects of the PhA assessment have raised interest. The reported relationship between low PhA values and age-related muscle depletion and its correlation with reduced muscle function (strength and endurance) assessed with dynamometry opens a new path in the value of determining PhA in the assessment of malnutrition and sarcopenia [19, 34, 35]. Emerging interest in monitoring PhA values as an indicator of inflammatory states and oxidative stress in obesity and metabolic diseases reinforces its use in patients with SARS-CoV2 infection as a factor in the evaluation of the prognosis of the patient [36].

Beyond the PhA as a primary measurement, we can analyse the raw bioelectrical data. In this sense, the height (H)-adjusted R and Xc results are standardized when compared to the population reference pattern. The standardization of the PhA adjusted by age and sex concerning population references allows us to compare different clinical populations [37]. In the same way, the analysis of the raw bioelectrical components R/H transformed into hydration status, and Xc/H transformed into nutrition status with their mean values and population standard deviation allow us to analyse segregated results of the hydration and nutrition components contained in the PhA [38, 39].

PhA and SPhA are BIA measurements that are novel options for practical assessment and clinical evaluation of impaired nutritional status and prognosis among hospitalized COVID-19 patients and could potentially contribute to enhanced patient care and clinical outcomes. The literature review reported that a lower PhA increased the odds of COVID-19 complications and mortality during variable period-time [14, 22, 23, 40–44].

**Fig. 2:**
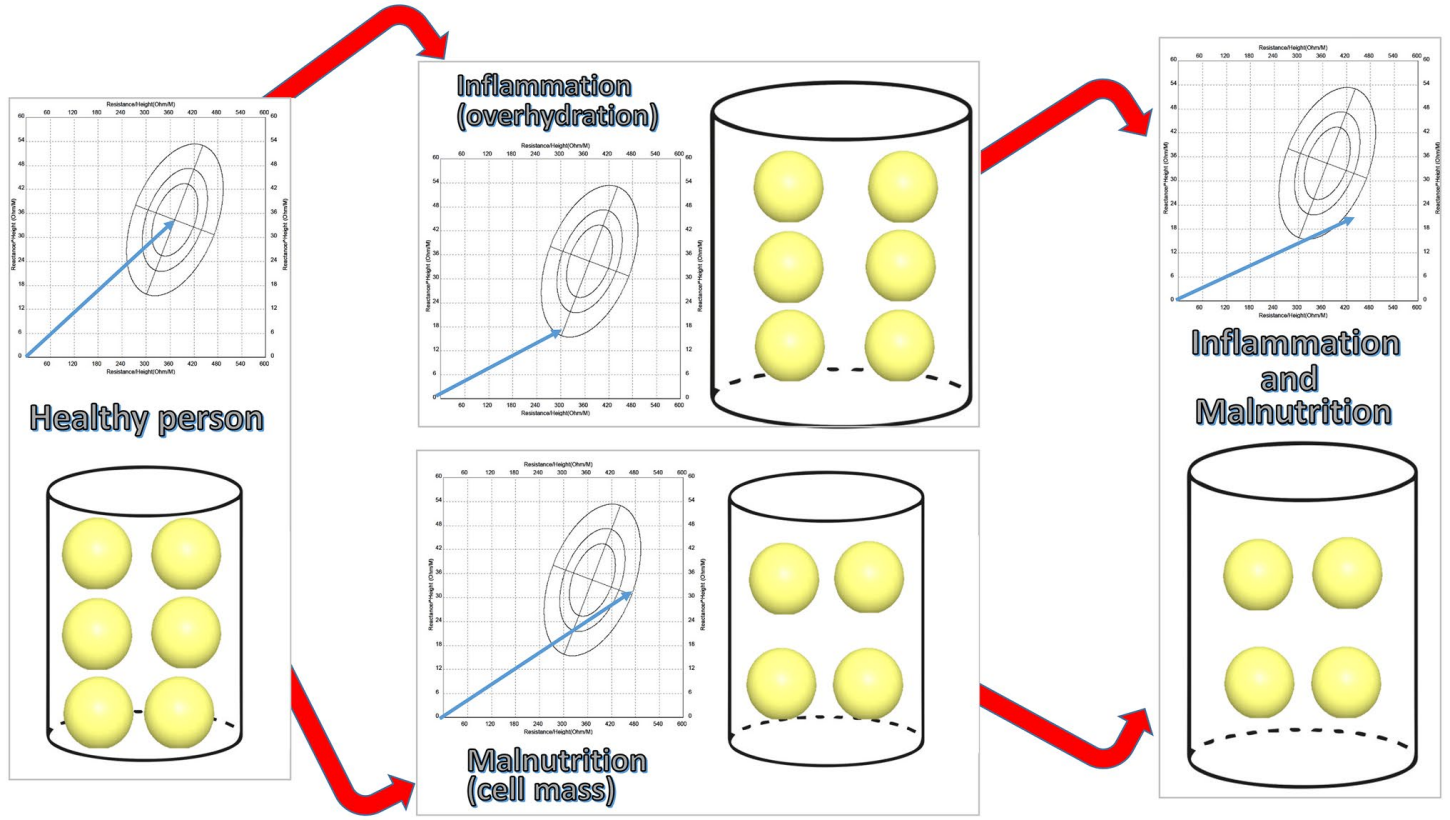
Interpretation of the bioelectrical component value of Phase Angle in COVID-19. PhA is a crude measurement. The analysis of its raw bioelectrical components R/H transformed into hydration status and Xc/H transformed into nutrition status allow us to analyse segregated results of the hydration/inflammation and nutrition components contained in the PhA. R: resistance; H: height; Xc: reactance; PhA: phase angle

In SARS-CoV2 patients, the interpretation of PhA requires careful consideration of the raw bioelectrical data to identify the R and Xc changes, due to both overhydration status associated with an increased inflammatory process and malnutrition status contributing to a decline in the PhA value [16, 22] (Fig. 2).

## Results

**Fig. 3:**
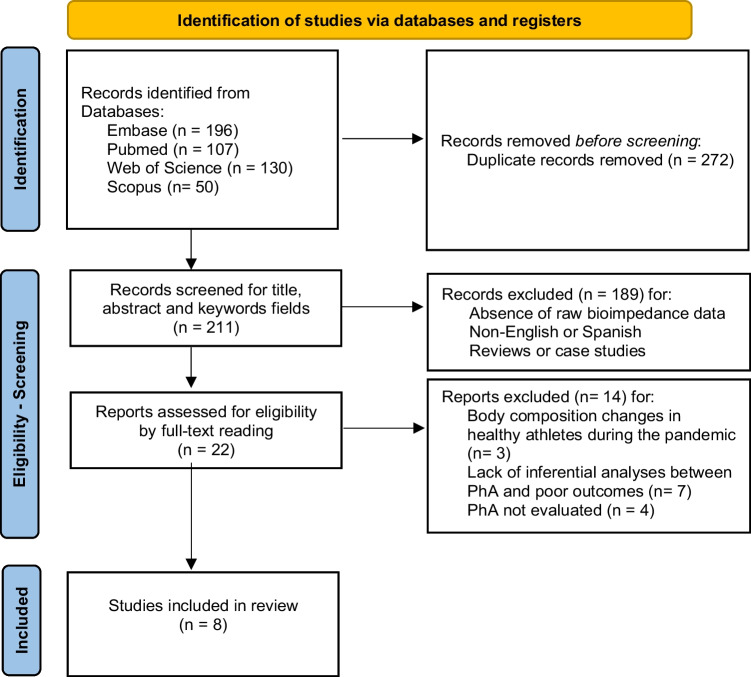
Flow-chart diagram for systematic review of PhA and SARS-CoV2 infections. PRISMA 2020

Our search procedure produced a total of 483 studies, as exposed in the flow-chart (Fig. 3). Of these articles identified from the databases, 272 were removed before the screening process through duplication. Based on our inclusion criteria (PICO) and exclusion criteria, the analysis of titles, keywords, and abstracts identified 22 potentially eligible studies. After reading the complete text, eight relevant studies were finally included in our systematic review of PhA and SARS-CoV2 infection [14, 22, 23, 40–44]. Fourteen reports were excluded due to lack of evaluation of PhA for clinical outcomes or relevance for SARS-CoV2 infection.

### Characteristics of the included studies

Eight studies were included, six were prospective observational cohort studies from one centre [22, 23, 40–42, 44], while one of them was multicentre with two referral centres [41], one was an observational cross-sectional cohort study [14] and other was a retrospective observational study [43]. Most of the studies were conducted in European countries [14, 22, 23, 43, 44] and Mexico [40–42] (Table 1).

**Table 1:** Characteristics of the studies, main outcomes studied and conclusions

**Variables**	**Moonen H.P. et al.** [14]	**Del Giorno R. et al.** [43]	**Cornejo-Pareja I. et al.** [22]	**Osuna-Padilla I.A. et al.** [42]	**Reyes-Torres C.A. et al.** [41]	**Moonen H.P. et al.** [23]	**Da Porto A. et al.** [44]	**Rosas-Carrasco O. et al.** [40]
Country	Netherlands	Switzerland	Spain	Mexico	Mexico	Netherlands	Italy	Mexico
Study Design	Observational Cross-sectional Study	Retrospective Observational Study	Prospective Observational Cohort Study	Prospective Observational Cohort Study	Prospective Observational Cohort Study	Prospective Observational Cohort Study	Prospective Observational Cohort Study	Prospective Observational Cohort Study
Participants centres	One referral centre	One referral centre	One referral centre	One referral centre	Two referral centre	One referral centre	One referral centre	One referral centre
Clinical profile	General ward and ICU patients	General ward patients	General ward and ICU patients	ICU patients	Post-extubated ICU patients	General ward and ICU patients	General ward patients	General ward patients
Patients (n, age, sex)	n = 54 67y (IQR 64–71) 63% male	n = 90 64.5y (SD ± 13.7) 67.8% male	n = 127 69y (IQR 59–80) 59.1% male	n = 67 55.3y (SD ± 13.6) 76% male	n = 112 54y (SD ± 12,0) 82% male	n = 150 68y (IQR 66–70) 67% male	n = 150 69y (IQR 58–78) 68.7% male	n = 104 62,7y (SD ± 15,0) 51.9% male
Outcomes and follow-up time	28d-Disease severity: ICU admission + complications or mortality	LOS (> 21d), in-hospital mortality and ICU admission, Appetite-loss (until discharge)	90d- Mortality	60-d Mortality	Dysphagia post-extubation (until ICU discharge)	90d-Disease severity: ICU admission + complications or mortality and LOS	60-d IMV, In-hospital mortality	20d- Mortality
Event rate	Disease severity: 34/54 Mortality: 8/54, Complications: 28/54, ICU admission: 24/54	LOS: 19/90, In-hospital mortality: 18/90, Appetite-loss: 28/90	16/127	25/67	46/112	ICU: 41/150 Complications: 59/150 Disease severity: 77/150	IMV: 23/150 Mortality: 22/150	42/104
Conclusion	A lower PhA increased the odds of morbidity, mortality and severe COVID-19	PhA does not seem to add further predictive value	Low PhA < 3.95° is a significant independent predictor of mortality risk in COVID‐19 (sensitive 93.8% and specific 66.7%)	Low PhA value is a biological marker that could be a predictor of 60‐d mortality in COVID-19 critically ill PhA < 3.85° (♀) and < 5.25° (♂) could need special nutrition attention	Low PhA was associated with dysphagia in post-extubated patients. Lower PhA was an independent factor for swallowing recovery at discharge	PhA is independently correlated with an adverse outcome of COVID-19	Malnutrition diagnosed by BIVA was associated with worse outcomes in COVID-19 patients. PhA was not significantly associated with need of IMV (only 0.7%) or mortality at 60 d	PhA was higher risk predictor of mortality than APACHE, SOFA, and CURB-65 at admission in patients hospitalized for COVID-19

*NA* not available, *SD* standard deviation, *IQR* interquartile range, *y* years, *d* days, *ICU* intensive care unit, *LOS* length hospital stay, *IMV* intensive mechanical ventilation, *APACHE* acute physiology and chronic health evaluation, *SOFA* sepsis-related organ failure assessment, *CURB-65* score for pneumonia severity

A total of 854 admission patients with SARS-CoV2 infection participated in our systematic review. In all the studies, the male sex was predominant (> 60%). The mean age of the European studies was higher, prevailing at a mean age of > 65 years, while the mean age in two of the Mexican studies [41, 42] was lower with a mean of 55 years, corresponding to studies focused on ICU patients (Table 1).

The BIA device used for the measurement were InBody S10®, a multifrequency model [14, 23, 41, 42], BIA 101 BIVA (Akern), a 50 kHz phase-sensitive model [22, 43], SECA® model mBCA 525, a multifrequency model [44], and BIA Quantum V RJL Systems, a phase-sensitive model [40] (Table 2). In general, the PhA measurements were carried out in the first 24-72 h of admission and follow-up time for adverse outcomes ranged from 20 to 90 d. The measurement technique was performed with patients in a supine position [22, 40–44] and using a frequency of 50 kHz [14, 22, 23, 40, 41] in most of the studies (Table 2).

**Table 2:** Bioelectrical parameters: measurement methodology and main result of the overall study sample and comparative groups

**Variables**	**Moonen H.P. et al.** [14]	**Del Giorno R. et al.** [43]	**Cornejo-Pareja I. et al.** [22]	**Osuna-Padilla I.A. et al. **[42]	**Reyes-Torres C.A. et al. **[41]	**Moonen H.P. et al.** [23]	**Da Porto A. et al.** [44]	**Rosas-Carrasco O. et al.** [40]
Measurement methodology (BI-device and procedures)	InBody S10® (InBody Co., Ltd., Seoul, Korea) (PhA: 50 kHz) Variable: 2-11d	BIA 101, Akern Bioresearch®, Florence, Italy (50 kHz) The first 24 h after admission	BIA 101, Akern (Bioresearch®, Florence, Italy) (50 kHz) The first 72 h of admission	InBody S10® (InBody Co,Ltd, Seoul,Korea) (Frequency NA) Within 48 h of ICU admission and IMV	InBody S10® (InBody Co., Ltd., Seoul, Korea) (PhA: 50 kHz) At first 24 h of postextubation	InBody S10® (InBody Co., Ltd., Seoul, Korea) (PhA: 50 kHz) The first 24 h of admission	SECA® (model mBCA 525; Seca gmbh & Co, Hamburg, Germany) (Frequency NA) The first 36 h after admission	BIA Quantum V, RJL Systems (50 kHz) The first 24 h of admission
Comparative groups	Ward vs. ICU patients	Normal vs. Nutritional risk patients	Survivors vs. Non-survivors	Survivors vs. Non-survivors	Non dysphagic vs. Dysphagic patients	Ward vs. ICU patients	Non-malnourished vs. Malnourished patients	Survivors vs. Non-survivors
PhA (mean and reference values, RV)	4.5º (4.2–4.8) RV: 5.6º-6.5º	5.6º (± 1.14) RV: not available	4.4º (3.2–5.4) RV: ♂:5.3–6.6º, ♀: 4.8-6º	5.0º (SD ± 1.2) RV: Low SPhA < -1.65	4.8º (SD ± 1.1) RV: Low PhA < 4.8º	5.4º (5.2–5.6) RV: 5.6º-6.5º	5.5º (± 1.5) RV: not avalaible	5.1º (± 1.6) RV: < 3.66º (-SD 1.65, p5)
PhA comparative groups	4.8º (4.4–5.2) vs. 4.1º (3.8–4.5), p = 0.017	5.7(± 4) vs. 5.4 (± 1.3), p = 0.386	4.5º (3.5–5.5) vs. 2.8º (2.08–3.68), p < 0.001	5.4º (± 1.2) vs. 4.4º (± 1.0), p < 0.001	5.2º (± 0.93) vs. 4.0º (± 0.96), p < 0.001	5.4º (5.2–5.7) vs. 5.2º (4.9–5.4), p = 0.14	5.9º (± 1.5) vs. 4.5º (± 0.7), p > 0.001	5.43º (± 1.5) vs. 4.8º (± 1.7), p = 0.0309
SPhA (mean, and RV)	Not available	Not available	-0.8 (-2.0 – 0.2)	-2.5 (-3.8 –-0.83)	Not available	Not available	Not available	Not available
SPhA comparative groups	Not available	Not available	-0.7 (-1.8 –0.3) vs. -2.95 (-3.6 – -1.3), p < 0.001	-3.7 (± 1.4) bs -2.0 (± 1.8), p = 0.002	Not available	Not available	Not available	Not available
Hydration status (mean and RV)	ECW/TBW: 0.40 (0.39–0.40) RV: 0.380	TBW/FFM: Not available	TBW/FFM: 73.8% (73.3–84.3) RV: 72.7–74.3%	ECW/TBW 0.39 (± 0.01) RV: not available	ECW/TBW 0.395 (± 0.138) RV: if > 0.380 overhydration	ECW/TBW: 0.39 (0.39–0.39) RV: 0.36–0.39	ECW/TBW: 0.451 (± 0.033) RV: not available	Not available
Hydration comparative groups	0.39 (0.39–0.40) vs. 0.40 (0.40–0.41), p = 0.015	74.3% (± 2.7) vs. 75.2% (± 3.2), p = 0.204	73.7% (73.2–82.1) vs. 85.2% (76.9–89.3), p < 0.001	0.386 (± 0.01) vs. 0.398 (± 0.01), p = 0.001	0.389 (± 0.010) vs. 0.402 (± 0.014), p < 0.001	0.39 (0.39–0.39) vs. 0.39 (0.39–0.39), p = 0.014*	0.444 (± 0.032) vs. 0.47 (± 0.027), p < 0.001	Not available
Cellular mass (mean and RV)	SMI: 8.0 kg/m^2^ (7.6–8.4) FFM: 59.2 kg (55.4–63.1) SLM: 55.9 kg (52.3–59.5)	FFM: 58.2 kg (± 10.7) FFM index: 34.3 kg/m (± 6.0) BCM: 17.8 kg/m (± 4.7)	BCM: 21.4 kg (16.3–27.9)	Not available	Not available	SMI: 8.1 kg/m^2^ (7.8–8.3) BCM: 37.7 kg (36.2–39.2) FFM: 58.5 kg (56.3–60.7) SML: 55.1 kg (53.1–57.2)	FFM index 59.1 kg/m^2^ (± 13.3) SMM index: 27.1 kg/m^2^ (± 8.4)	AMM index: 7.44 kg/m^2^ (± 1.51)
Cellular mass comparative groups	SMI: 7.5 (7.1–8.0) vs. 8.6 (7.9–9.2), p = 0.006 FFM: 55.5 (50.7–60.3) vs. 63.9 (57.8–70.0), p = 0.028 SLM: 52.4 (47.9–56.9) vs. 60.3 (54.6–66.1), p = 0.028	FFM: 12.4 (± 5.3) vs. 12.5 (± 5.93), p = 0.921 FFM index: 33.9 (± 4.9) vs. 35.8 (± 9.4), p = 0.253 BCM: 17.6 (± 3.9) vs. 18.9 (± 6.9), p = 0.297	BCM: 23 (18.5–31.5) vs. 14.2 (10.2–18.0), p < 0.001	Not available	Not available	SMI: 7.9 (7.7–8.2) vs. 8.4 (8.1–8.8), p = 0.028 BCM: 36.8 (35.2–38.6) vs. 40.0 (37.8–42.0), p = 0.026 FFM: 57.2 (54.8–60.0) vs. 61.8 (58.6–65.0), p = 0.026 SML: 53.9 (51.7–56.4) vs. 58.3 (55.3–61.3), p = 0.024	FFM index: 62.1 (± 13.1) vs. 49.9 (± 9.3), p < 0.001 SMM index: 29.1 (± 8.2) vs. 20.9 (± 5.7), p < 0.001	AMMI: 7.25 (± 0.92) vs. 6.03 (± 1.29), p = 0.0148
R	Not available	Not available	R/H 302.5 Ω/m (272.2–366.3)	Not available	Not available	Not available	Not available	Not available
Resistance comparative groups	Not available	Not available	301.7 (272.2–363.5) vs. 334.6 (251.5–370.3), p = 0.769	Not available	Not available	Not available	Not available	Not available
Reactance	Not available	Not available	24.7 Ω/m (16.3–31.1)	Not available	Not available	Not available	Not available	Not available
Reactance comparative groups	Not available	Not available	25.3 (18.9–32.4) vs. 15.0 (10.5–22.6), p = 0.001	Not available	Not available	Not available	Not available	Not available

*NA* not available, *SD* standard deviation, *IQR* interquartile range, *ICU* intensive care unit, *RV* reference values determined as usual, *PhA* phase angle, *ECW* extracellular water, *TBW* total body water, *FFM* fat-free mass, *SMI* skeletal muscle mass index, *SLM* soft lean mass, *SMM* skeletal muscle mass, *AMMI* appendicular muscle mass index, *R* resistence, *Xc* Reactance

^*^Results published in the original article, with incongruence between the described values of hydration and the significant statistical differences found

### Findings

The average PhA ranged from 4.4 to 5.6º. The lowest PhA values were recorded in the studies that included patients with the most severe SARS-CoV2 infection (ICU patients) [14, 22, 41, 42]. The studies focused on COVID-19 patients admitted to general ward had higher mean PhA values [40, 43, 44], than those involving patients admitted to the ICU [14, 22, 41, 42]. In all studies, significantly lower PhA values were found in patients with poor-outcome compared to the comparison groups, except Moone et al. [23] and Del Giorno et al. [43] studies that reported no significant differences between general ward and ICU patients and patients with and without nutritional risk, respectively (Table 2).

A decrease in R and Xc both contribute to the overhydration state for an increase in the inflammatory process and the malnutrition state that contributes to a decrease in PhA value. The publications analysed raw bioelectrical parameters such as R, Xc, and SPhA, as well as parameters related to hydration-inflammation status and those related to cell mass and nutritional status (Table 2). These raw bioelectrical measurements were only analysed in one of the studies [22], while the SPhA value was described in two of the studies [22, 42]. The principal finding was significant differences between the comparative groups (survivors vs. non-survivors) for all studies. Importantly, the mean SPhA was -2.5 (i.e., 2.5 SD less than the healthy population of his age and sex) in critically ill patients while in general ward patients, it was -0.8 (i.e., 2.5 SD less than the healthy population of his age and sex). Thus, patients with more severe disease (critically ill patients) had SPhA further away from the reference population compared to patients with more stable disease (general hospital ward) (Table 2).

Seven studies investigated the hydration status of the patients with COVID-19. Five studies [14, 23, 41, 42, 44] reported hydration status as the ECW/TBW by BIA, and two reports provided [22, 43] the TBW/FFM ratio by BIA. The mean hydration status ranged from 0.39 to 0.45 for EWC/TBW and 73.8% for TBW/FFM. Significant differences were detected in the hydration status of the comparative groups analysed [14, 22, 23, 41, 42, 44]. However, Del Giorno et al. [43], found no significant differences in hydration status between COVID-19 patients with and without nutritional risk (Table 2).

Various assessments of lean soft tissue mass compared in the studies [14, 22, 23, 40, 43, 44] (Table 2). Moonen et al. [14, 23] identified depletion in indices of muscle and FFM between ICU and general ward patients. Significant reductions in BCM [22, 40], AMMI [40] and FFMI and SMMI [44] were found between comparison control groups (survivors vs. non-survivors, dysphagic vs non-dysphagic patients and malnourished vs. non-malnourished patients). However, Del Giorno et al. [43] found no significant differences between the study groups (Table 2).

### Poor outcomes researched in admission patients with COVID-19

The principal outcomes under analysis were mortality [14, 22, 40, 42–44] and disease severity, defined as the need for mechanical ventilation or a composite score, including the need for ICU admission [14, 23, 43, 44]. The presence of complications, such as thrombo-embolic event, renal failure, delirium, pulmonary fibrosis, dysphagia post-extubating [14, 23, 41] and LOS [23, 43] constituted additional poor outcomes (Table 1).

(I) Mortality
  The studies that explored the association of PhA as an independent prognostic factor for mortality used the odds ratio (OR) or hazard ratio (HR) analysis. The highest HR observed for 90-d mortality was 3.912 [95% CI (1.322–11.572), p = 0.014] in an adjusted model for sex, age, BMI, comorbidities, and hydration status in 127 COVID-19 patients (ICU and general ward admission) [22]. The PhA value cut-off point for predicting mortality was 3.95º with a sensitivity of 93.8% and specificity of 66.7% [AUC = 0.839, p = 0.001] [22].
  In an adjusted model by sex, age, comorbidities, prognostic scales (CURB-65 and SOFA), and AMMI in 104 hospitalized patients followed up for 20-d, a low PhA < 3.66º had an HR = 2.571[95% CI (1.217–5.430), p = 0.013] [40]; while an adjusted model including nutrition risk (NUTRICscore) and age in 67 critical ill COVID-19 patients demonstrated 60-d mortality HR = 3.08 [95% CI (1.12–8.41), p = 0.02] [42]. Further, a PhA < 5.25° [AUC = 0.74, 95% CI (0.60–0.88), p = 0.003) for males and < 3.85º [AUC = 0.83, 95% CI (0.60–0.99), p = 0.03) in females were predictive mortality markers with a sensitivity (72% and 66.7%, respectively) and specificity (72% and 90%, respectively) [42]. In 54 hospitalized COVID-19 patients (ICU and general ward), a higher PhA value was associated with a significantly lower risk of 28-d mortality [OR: 0.208, p = 0.025] [14].
(II) Length of stay (LOS)
  A greater PhA was also related to a lower hospital LOS [OR = 0.875, 95% CI (0.765–1.001), p = 0.037] [23] in a population of 150 patients hospitalized in the ICU and general ward. Osuna-Padilla et al. also found that lower PhA was associated with a longer LOS [r = −0.33, p = 0.03] without deepening multivariate analysis with adjusted models [42]. However, in the study of 90 hospitalized COVID-19 patients investigators did not find a similar association [OR = 1.04, 95%CI (0.12–8.63), p = 0.974] [43].
(III) Severity of disease
  Phase angle adjusted for age, sex and BMI was significantly associated with the need to IMV [HR = 1.007, 95% CI (0.714–1.422), p = 0.007] in 150 hospitalized ward patients [44]. Similarly, Osuna-Padilla et al. found a significant negative correlation between PhA and IMV duration [r = −0.42, p = 0.005] without exploring multivariate analyses with adjusted models [42].
  Other studies of patients on the ICU and general ward support the prognostic value of PhA. Among 150 patients, a higher PhA was associated with a lower rate of admission to the ICU [OR = 0.531, 95% CI (0.285–0.989), p = 0.021] [23]. PhA adjusted for age, sex and BMI was significantly associated with IMV [HR = 1.007, 95% CI (0.714–1.422), p = 0.007] in hospitalized ward patients [44]. In the composite score studies referred to in the mortality section, PhA was significantly related to the severity of disease in Moonen et al. studies [14, 23], while Del Giorno et al. [43] found no significant differences.
  PhA also was as an independent prognostic factor with a composite outcome such as ICU admission and complications including mortality [OR 0.299, p = 0.046] [14] and [OR = 0.502, 95% CI (0.281–0.898), p = 0.015), respectively [23]. While Del Giorno et al. found no association between PhA and the composite outcome that includes ICU admission and in-hospital mortality [OR = 0.59, 95%CI (0.21–1.71), p = 0.332] [43]. Likewise, the PhA showed not associated with mortality in 150 ward patients into an adjusted model by age, sex and BMI [HR = 1.084, 95% CI (0.803–1.463), p = 0.081] [44].
(IV) Complications
  Among 150 patients on an ICU and ward hospitalized patients, a lower PhA was a significant predictor of complications such as thrombo-embolic event, renal failure, delirium, and pulmonary fibrosis [OR = 0.579, 95% CI, (0.344–0.973), p = 0.031] [23]. In contrast, PhA was not a significant prognostic factor for these complication in 54 patients [OR = 0.413, p = 0.061], but PhA was a significantly predictive of severe disease or mortality, as previously shown [14].
  Post-extubating dysphagia is emerging as a complication of SARS-CoV2 infection. Among 112 critically ill patients, a PhA < 4.8º was identified as an independent predictor of post‐extubating dysphagia in a model adjusted for age and sex [OR = 12.2, 95% CI(4.3–34.1), p < 0.05] [41]. This initial finding suggests a mechanism by which low PhA can contribute to malnutrition in patients with SARS-CoV2 infection.
  Studies using regression analysis to determine the significance of the PhA as a predictive marker of poor outcomes included various independent variables for model development. At the same time, age and sex were employed homogeneously in all studies [14, 22, 23, 40–44], most used an indirect indicator of body composition such as BMI [14, 22, 23, 43, 44] or AMMI [40]. Other studies introduced nutritional risk scales such as NUTRIC score [42], hydration status [22], or cell mass [40]. Only three studies included comorbidities [22, 40, 43] as possible confounding factors, and four studies used the risk or prognostic scales [14, 23, 40, 43], making these analyses heterogeneous. This leads to the fact that there may be some differences in the results among some of the studies evaluated, as described previously in this section.

### Quality of studies

The initial literature review yielded 483 publications, only 8 publications covered all four topics related to PICO issues. The quality of the evidence was evaluated following the GRADE method (Table 3), which allowed the scientific committee to make 4 evidence-based recommendations on the prognostic and clinical value of PhA measurements (Table 4).

**Table 3:** GRADE evidence in SARS-CoV2 infection: Summary of the body of evidence, the judgments about the quality of the evidence, key results, and importance **Question**: Prognostic value of Low Phase Angle in patients with SARS-CoV2 infection during short- and medium-term follow-up **Setting**: General ward and ICU admitted patients with SARS-CoV2

**№ of studies**	**Certainty assessment**						**Effect**			**Certainty**	**Importance**
	**Study design**	**Risk of bias**	**Inconsistency**	**Indirectness**	**Imprecision**	**Other considerations**	**№ of events**	**№ of individuals**	**Rate (95% CI)**		
**All-cause mortality** in patients (ward and ICU hospitalized SARS-CoV2 infection patients) (follow-up: 28 days; assessed with: phase angle)											
1 Moonen 2020	observational studies	serious^a^	not serious	not serious	serious^b^	None	8	54	event rate 0.2 (-- to --)	⨁⨁◯◯ Low	CRITICAL
**All-cause mortality** in patients ( ward and ICU hospitalized SARS-CoV2 infection patients) (follow-up: 90 days; assessed with: phase angle)											
1 Cornejo-Pareja 2021	observational studies	not serious	not serious	not serious	serious^c^	None	16	127	event rate 3.9 (1.322 to 11.572)	⨁⨁⨁◯ Moderate	CRITICAL
**All-cause mortality** in patients (ICU hospitalized SARS-CoV2 infection patients) (follow-up: 60 days; assessed with: phase angle (Low SPhA < -1.65)											
1 Osuna-Padilla 2021	observational studies	not serious	not serious	serious^d^	not serious	None	25	67	event rate 3.1 (1.12 to 8.41)	⨁⨁⨁◯ Moderate	CRITICAL
**All-cause mortality** in patients (General ward hospitalized SARS-CoV2 infection patients) (follow-up: 60 days; assessed with: phase angle)											
1 Da Porto 2021	observational studies	not serious	not serious	serious^e^	serious^f^	None	22	150	event rate 1.1 (0.803 to 1.463)	⨁⨁◯◯ Low	CRITICAL
**All-cause mortality** in patients (General ward hospitalized SARS-CoV2 infection patients) (follow-up: 20 days, assessed with: phase angle < 3.66º)											
1 Rosas-Carrasco 2022	observational studies	serious^g^	not serious	not serious	not serious	None	42	104	event rate 2.6 (1.217 to 5.430)	⨁⨁⨁◯ Moderate	CRITICAL
**Prolonged length hospital stay** (defined as length hospital stay > 21 days) in patients (General ward hospitalized SARS-CoV2 infection patients) (assessed with: phase angle)											
1 Del Giorno 2020	observational studies	serious^h^	serious^i^	not serious	serious^j^	None	19	90	event rate 1.0 (0.12 to 8.63)	⨁◯◯ Very Low	IMPORTANT
**Length of hospital stay** in patients (General ward and ICU hospitalized SARS-CoV2 infection patients) (follow-up: 90 days; assessed with: phase angle)											
1 Moonen 2021	observational studies	not serious	not serious	not serious	seriou^k^	None	–	150	event rate 0.9 (0.765 to 1.001)	⨁⨁◯◯ Low	IMPORTANT
**Severity disease** (evaluated by a composite score that comprised ICU admission and complications including mortality) in patients (General ward and ICU hospitalized SARS-CoV2 infection patients) (follow-up: 28 days; assessed with: phase angle)											
1 Moonen 2020	observational studies	serious^a^	not serious	not serious	serious^bl^	None	34	54	event rate 0.3 (-- to --)	⨁⨁◯◯ Low	CRITICAL
**Severity disease** (evaluated by a composite score that comprised ICU admission and complications including mortality) in patients (General ward and ICU hospitalized SARS-CoV2 infection patients) (follow-up: 90 days; assessed with: phase angle)											
1 Moonen 2021	observational studies	not serious	not serious	not serious	serious^c^	None	77	150	event rate 0.5 (0.281 to 0.898)	⨁⨁⨁◯ Moderate	CRITICAL
**Severe Disease** (defined as Need for supportive care) with intensive mechanical ventilation in patients (General ward hospitalized SARS-CoV2 infection patients) (follow-up: 60 days; assessed with: phase angle)											
1 Da Porto 2021	observational studies	not serious	not serious	serious^e^	serious^f^	None	23	150	event rate 1.0 (0.714 to 1.422)	⨁⨁◯◯ Low	IMPORTANT
**Severity disease** (evaluated by composite score that comprised in-hospital mortality and needs ICU admission) in patients (General ward hospitalized SARS-CoV2 infection patients) (assessed with: phase angle)											
1 Del Giorno 2020	observational studies	serious^h^	serious^m^	not serious	serious^n^	None	18	90	event rate 0.6 (0.21 to 1.71)	⨁◯◯◯ Very Low	CRITICAL
**COVID-19 complications** (defined as thrombo-embolic event, renal failure, delirium, among others) in patients (General ward and ICU hospitalized SARS-CoV2 infection patients) (follow-up: 28 days; assessed with: phase angle)											
1 Moonen 2020	observational studies	serious^a^	not serious	not serious	serious^b^	None	28	54	event rate 0.4 (-- to --)	⨁⨁◯◯ Low	IMPORTANT
**COVID-19 complications**, (defined as thrombo-embolic event, renal failure, delirium, lung fibrosis, among others) in patients (General ward and ICU hospitalized SARS-CoV2 infection patients) (follow-up: 90 days; assessed with: phase angle)											
1 Moonen 2021	observational studies	not serious	not serious	not serious	serious^c^	None	59	150	event rate 0.6 (0.344 to 0.973)	⨁⨁⨁◯ Moderate	IMPORTANT
**COVID-19 complications** (defined as dysphagia post-extubation) in patients (ICU hospitalized SARS-CoV2 infection patients) (assessed with: phase angle < 4.8º)											
1 Reyes-Torres 2021	observational studies	not serious	not serious	not serious	serious^c^	None	46	112	event rate 12.2 (4.3 to 34.1)	⨁⨁⨁◯ Moderate	IMPORTANT

**Explanations:**

^a^Cross-sectional study. No predefined criteria for the PhA measurement at admission and short follow-up time –28d–

^b^Not report confidence interval

^c^Wide confidence intervals

^d^Indirect evidence: only assessed ICU patients. Applicability may not be the same in overall COVID-19 hospitalized patients. Populations may differ in varying degrees of disease severity

^e^Risk results associated with malnutrition are found, but not significant concerning to mortality or the need for supportive care with intensive mechanical ventilation

^f^When performing Cox regression (multivariate) analysis with an adjusted model (with possible confounders) significance is lost. The confidence interval includes the null value

^g^To assess overall mortality, it includes a short follow-up time –20d–

^h^Retrospective Observational Study

^i^Heterogeneity between results: the significant result for the study of PhA in predicting prolonged length of hospital stay (defined by > 21 days) assessed by receiver operating characteristic (ROC) curve, but when performing Logistic regression (multivariate) analysis significance is lost

^j^It not provides PhA cut-off points in the analysis of receiver operating characteristic (ROC) curves for the length of hospital stay. It not defines why it establishes in 21 days the point to define prolonged length hospital stay

^k^Wide confidence interval, which crosses the value of 1

^l^When performing Logistic regression (multivariate) analysis with an adjusted model (with possible confounders) significance is lost

^m^Heterogeneity between results: with the significant result for the study of PhA in predicting severity disease by composite outcomes assessed by receiver operating characteristic (ROC) curve, but when performing Logistic regression (multivariate) analysis significance is lost

^n^It not provide PhA cut-off points in the analysis of receiver operating characteristic (ROC) curves for severity disease. And a wide confidence interval, which crosses the value of 1

**Table 4:** Evidence-based recommendations following the GRADE method for hospitalized patients with SARS-CoV2 infection

**No.**	**Topic**	**Strength of recommendation**	**Quality of evidence**	**Recommendation**
R1	Phase angle	Strong	Moderate	The phase angle, measured by bioelectrical impedance analysis, can be used for predicting mortality in hospitalized patients with SARS-CoV2
R2	Phase angle	Weak	Very Low-Low	The phase angle, measured by bioelectrical impedance analysis, can be suggest a longer length of hospital stay in hospitalized patients with SARS-CoV2
R3	Phase angle	Weak	Very Low-Low	The phase angle, measured by bioelectrical impedance analysis, can advise severe disease in hospitalized patients with SARS-CoV2
R4	Phase angle	Strong	Low–Moderate	The phase angle, measured by bioelectrical impedance analysis, can be used for predicting complications in hospitalized patients with SARS-CoV2 infection

PhA can be used, with a strong recommendation strength and moderate evidence quality, to predict mortality in hospitalized patients with SARS-CoV2 infection. Similarly, PhA, which has a weak strength of recommendation and a very low-low quality of evidence, can predict a longer LOS hospital stay and advise increased risk of severe disease in hospitalized patients with SARS-CoV2 infection. Also, PhA, with a strong strength of recommendation and a low to moderate quality of evidence, can be used to predict complications in hospitalized patients with SARS-CoV2 infection.

### Usefulness of the PhA as a prognostic factor of poor outcomes: meta-analysis

**Fig. 4:**
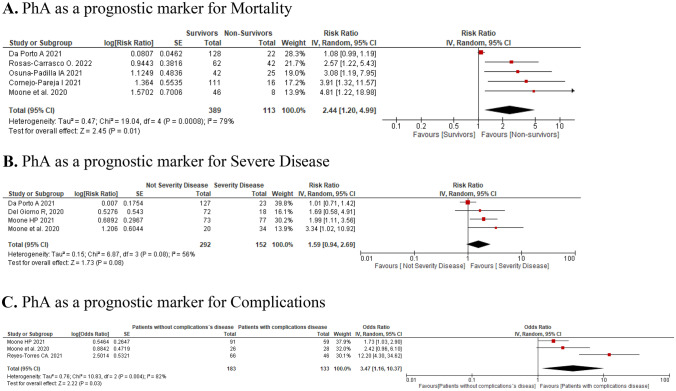
The subgroups analyses of PhA as a prognostic marker for poor outcomes in hospitalized COVID-19 patients. The severity data of OR or HR and 95% CI from 8 studies were pooled in this meta-analysis and the result of the meta-analysis was described as a forest plot. 8 studies were grouped, in the main poor outcomes studied: mortality (**A**), severity disease (**B**) and complications (**C**). OR: Odds ratio; HR: Hazard ratio; CI: confidence interval; intensive care unit

A randomized-effect or fixed-effect model used when the tests were characterized as heterogeneous or homogeneous, respectively, was employed for meta-analysis. Meta-analysis of data from 502 patients indicated a significantly increased mortality risk in COVID-19 patients with lower PhA [RR: 2.44; 95% CI (1.20–4.99), p = 0.01]. Heterogeneity between studies: I^2^ = 79% (p = 0.0008)]. A significantly increased complications risk was found in 316 COVID-19 patients with lower PhA [OR: 3.47, 95% CI (1.16–10.37), p = 0.03; Heterogeneity between studies: I2 = 82% (p = 0.004)]. Nevertheless, PhA was not a significant predictor of severe disease with 444 patients included [RR: 1.59, 95% CI (0.94–2.69), p = 0.08] (Fig. 4).

## Discussion

The present systematic review with meta-analysis evaluated the predictive capability of PhA on the clinical prognosis of COVID-19 patients. The overall result showed that a low PhA in hospitalized COVID-19 patients could predict a higher risk of death or complications. This systematic review was the first report of the combined use of the GRADE method and incorporating meta-analysis for the evaluation of the value of PhA as a prognostic marker for poor outcomes in hospitalized COVID-19 patients [45]; allowing statistical evaluation of the included studies and to analyse their quality to generate recommendations.

The reported PhA and other bioelectrical parameter values differed significantly in hospitalized COVID-19 patients between comparison groups. A low PhA was associated with the patient group, which had a poor clinical evolution or a severe disease with complications (critically ill, presence of complications or mortality). The decreased mean PhA values of the patients with COVID-19 are consistent with the findings of patients with other pathologies, such as inflammatory or infectious pathologies [46, 47], pulmonary disease [48], or patients admitted to the ICU [49].

When assessing the association between PhA and mortality risk of hospitalized patients with SARS-CoV2 infection, this analysis included five studies and 502 patients and determined a significant association. This finding was also seen in patients who were critically ill or had cancer, chronic kidney disease, or heart failure [21]. Also, the present review determined an association between low PhA and complications in hospitalized patients with SARS-CoV2 infection using data from three studies and 316 patients. A similar observation was reported in patients undergoing lung cancer surgery [50]. The severity of the disease was also related to low PhA values in liver disease [51]. Other studies discovered that a reduced PhA was predictive of more extended hospital stay in patients hospitalized in internal medicine [25] or medical and surgical patients in general ward [35]. Our systematic review found no significant association for PhA as a predictor of disease severity (four studies and 444 patients).

The inconsistency between the findings of our meta-analysis review and some individual reports may be due to the few studies that evaluated the association of PhA and poor outcomes in COVID-19 patients and specific technical characteristics and differences between studies. Studies were homogeneous for age (overall > 65 y), sex (male gender dominant > 60%) and clinical profile (e.g., hospitalized COVID-19 patients: ICU or general ward). However, they were heterogeneous relative to experimental design (e.g., prospective cohort study (most), cross-sectional study or retrospective study), follow-time (20–90 d), PhA measurement time (24-72 h after admission), BIA device (e.g., BIA InBody S10®; BIA 101 BIVA AKERN®; BIA SECA® model mBCA 525; BIA Quantum V RJL Systems®), and reference PhA values determined as usual. The PhA values were determined with substantially different BIA devices. Measurements were performed using different types of electrodes (gel wet electrodes vs dry contact), different BI technologies (single 50 kHz frequency phase-sensitive devices optimized calibration vs bioelectrical impedance spectroscopy (BIS) with phase sensitivity using phase detection mediated between 4 to 1000 kHz), and most of the studies did not specify the body position during measurement (recumbent vs standing). Use of different BIA devices to determine PhA (50 kHz compared to multifrequency) can influence the reported values [21]. PhA values are maximal near 50 kHz when measured directly and can vary if determined indirectly (e.g., modelled). Although PhA was measured at 50 kHz in most studies, not all studies specified the frequency at which they had measured PhA.

Thus, another significant matter is related to the PhA cut-off or the reference values employed in the analyses of the studies. Currently, there is no known specific and valid PhA value to identify mortality, severe disease or complications in COVID-19 patients. However, the lower PhA percentiles found in the studied groups were used as a cut-off. This problem of lack of reference values in the disease can be partially solved by analysing the SPhA. The age- and sex-adjusted SPhA may be useful to obtain a prognostic value. Thus, in two of the studies analysed in this systematic review, data on SPhA in relation to mortality are provided. SPhA is also associated with poor outcomes in other diseases [24].

The human body may be considered as a network of resistors (R), represented by body fluids and their electrolytes, and capacitors (Xc) consisting of cell membranes and tissue interfaces. Thus, BIA and PhA measurements may indicate fluid distributions (E/I) and cell mass (BCM). The preponderance of the studies of patients with COVID-19 identify simultaneously identify poor outcomes with low PhA values, and a common finding also overhydration status associated with a high degree of inflammation and a lower cell mass related to malnutrition and sarcopenia (Table 2).

Observational reports identify excess fluid accumulation and acral and pulmonary oedema in SARS-CoV-2 infection, especially those patients that progress to acute respiratory distress syndrome [52]. In SARS-CoV-2 infection, overhydration refers to an imbalance in fluid distribution between extracellular and intracellular water volumes with the expansion of ECW associated with systemic inflammation and aggressive fluid administration [53]. The pathogenesis of overhydration is due to the inflammatory component of the disease and the primary fluid retention due to cardiac or renal hemodynamic failure. This mechanism may be similar to the PhA changes that occur in heart failure [54] or kidney failure [55]. Therefore, patients with overhydration status are related with poor outcomes such as, a higher incidence of sepsis or complications with multiple organ failure constitutes inflammatory settings favourable to more fluid retention.

Similarly, patients with acute SARS-CoV2 infection tend to lose weight due to cachexia with catabolic and metabolic alterations that directly impact nutritional status [56]. Obesity is a risk factor for adverse outcomes of COVID-19[57]. It is associated with impaired measures of the quantity and quality of muscle mass and fat mass that exacerbate outcomes in severe SARS-CoV2 infection and can indirectly promote malnutrition [34, 58], which is highly related to mortality [35].

## Limitations and strength

Heterogeneity in studies is a main limitation of this systematic review. Body composition, sex, or age influence PhA values. Whereas control of these contributing factors is important in studies of potential biomarkers of prognosis, clinical and medical circumstances may not fully allow the avoidance of them. Also, confounding factors such as comorbidities or risk scales [59, 60] may be impractical to control. Statistical analytical methods allow for adjustment in age and gender and body composition, generally BMI, in the assessment of risk.

Similarly, adjustment for indices of nutritional status assessment and prognostic scales is infrequently performed in risk assessments.

The strengths of the systematic review were consistent with the PRISMA statement. This was achieved by using a rigorous research protocol to evaluate relevant publications, allowing adequate eligibility criteria and uniform search strategies to be used. The search utilized different databases and was reviewed by various authors; there were no restrictions made for year of publication or also not the place of execution. Also, this review used the GRADE method, which is a validated tool for the analysis of the quality of the evidence. GRADE proposes specific criteria that should be considered, particularly in observational studies.It provides guidance to describe clinical recommendations about the usefulness of PhA as a predictor of poor outcome markers. The meta-analysis enables the derivation of global results to determine risks of reduced PhA associated with mortality, severity, and complications of SARS-CoV2 infection.

## Conclusion

This systematic review determined that PhA, by BIA, is a valid prognostic indicator of mortality and complications in hospitalized COVID-19 patients.

Although the results are promising, there is still a deficiency of knowledge about the use of thresholds of the PhA in this population. Future studies are needed to identify PhA cut-off to guide therapeutic decisions more precisely. The reduction in values of PhA can indicate poor outcomes and allow a more adjusted supportive treatment of these patients.

## Abbreviations

AMMI: Appendicular muscle mass index
BI: Bioelectrical impedance
BIA: Bioimpedance analysis
BIS: Bioelectrical impedance spectroscopy
BMI: Body mass index
CI: Confidence interval
COVID-19: Coronavirus Disease 2019
ECW: Extracellular water
FFM: Fat-free mass
GRADE: Grading of Recommendations Assessment, Development and Evaluation
HR: Hazard ratio
ICU: Intensive Care Unit
IMV: Intensive Mechanical ventilation
LOS: Length of stay
OR: Odds ratio
PhA: Phase angle
PICO: Patient, Intervention, Comparison, Outcome
PRISMA: Preferred Reporting Items for Systematic Reviews
R: Resistance
RR: Risk Ratio
SARS-CoV-2: Severe acute respiratory syndrome coronavirus 2
SMI: Skeletal muscle mass index
SML: Soft lean mass
SMM: Skeletal muscle mass
TBW: Total body water
Xc: Reactance
